# The Progression of Hemophilic Arthropathy: The Role of Biomarkers

**DOI:** 10.3390/ijms21197292

**Published:** 2020-10-02

**Authors:** Gianluigi Pasta, Salvatore Annunziata, Alberto Polizzi, Laura Caliogna, Eugenio Jannelli, Alessandro Minen, Mario Mosconi, Francesco Benazzo, Matteo Nicola Dario Di Minno

**Affiliations:** 1 Department of Orthopaedics and Traumatology, Fondazione Policlinico IRCCS San Matteo, University of Pavia, 27100 Pavia, Italy; gianluigipasta@yahoo.it (G.P.); alberto.polizzi90@gmail.com (A.P.); l.caliogna@smatteo.pv.it (L.C.); eugenio.jannelli@libero.it (E.J.); alessandro.minen@gmail.com (A.M.); mario.mosconi@unipv.it (M.M.); fbenazzo@unipv.it (F.B.); 2 Department of Medical Sciences, Federico II University, 80131 Naples, Italy; dario.diminno@hotmail.it

**Keywords:** hemophilia, arthropathy, biomarkers

## Abstract

Background: Hemophilia A and B are X-linked congenital bleeding disorders characterized by recurrent hemarthroses leading to specific changes in the synovium and cartilage, which finally result in the destruction of the joint: this process is called hemophilic arthropathy (HA). This review highlights the most prominent molecular biomarkers found in the literature to discuss their potential use in the clinical practice to monitor bleeding, to assess the progression of the HA and the effectiveness of treatments. Methods: A review of the literature was performed on PubMed and Embase, from 3 to 7 August 2020. Study selection and data extraction were achieved independently by two authors and the following inclusion criteria were determined a priori: English language, available full text and articles published in peer-reviewed journal. In addition, further articles were identified by checking the bibliography of relevant articles and searching for the studies cited in all the articles examined. Results: Eligible studies obtained at the end of the search and screen process were seventy-three (73). Conclusions: Despite the surge of interest in the clinical use of biomarkers, current literature underlines the lack of their standardization and their potential use in the clinical practice preserving the role of physical examination and imaging in early diagnosis.

## 1. Introduction

Hemophilia A and B are X-linked congenital bleeding disorders caused by the absence or dysfunction of clotting factor VIII (FVIII) or factor IX (FIX), respectively. The degree of clotting factor deficiency influences the phenotype and spontaneous bleedings occurring into joints and muscles represent the most common clinical manifestation, mainly in the severe form of the disease (FVIII/FI < 1 IU/dL) [1].

The predilection for bleeding into large synovial joints is probably a consequence of the rich vascularization of synovial tissue and its exposure to intensive mechanical forces in combination with a shifted hemostatic balance [2,3]. Although the incidence of joint bleeding has been significantly reduced over the last 40 years through the extensive use of prophylaxis and clotting factor replacement, patients with hemophilia are still at risk for joint dysfunction due to bleeding.

Recurrent hemarthroses lead to specific changes in the synovium and cartilage, which finally result in destruction of the joint. This process is called hemophilic arthropathy (HA) [4]. The HA is associated with chronic pain and limited daily functioning that have a big impact on the quality of life in hemophilic patients [5].

Pathogenetic mechanism of HA is multifactorial and includes degenerative cartilage-mediated and inflammatory synovium-mediated components. Intra-articular blood leads to the formation of iron-catalyzed destructive oxygen metabolites resulting in chondrocyte apoptosis. This process has a negative effect on cartilage, subsequently the intra-articular blood affects the synovium, in addition to hemosiderin-induced synovial triggering. Intra-articular blood derived hemosiderin deposits are thought to be critical in the early phase of HA through the induction of a proliferative disorder with chronic synovitis, which then extends to the articular cartilage surface and ultimately resulting in destructive arthropathy. Both processes occur simultaneously, and while they influence each other, they probably do not depend on each other [4,6]. This model resembles the degenerative joint damage found in osteoarthritis (OA) as well as the inflammatory processes in rheumatoid arthritis (RA) [7].

The usual diagnostic methods of HA include plain radiography, ultrasonography (US) and magnetic resonance imaging (MRI) [8]. Developing diagnostic tools to identify patients at high risk of early appearance or progression of joint damage remains an important yet challenging aspect. Therefore, there is a big effort to identify a tool reflecting early dynamical changes in the joint. This tool may be represented by biochemical markers (in serum, urine or synovial fluid). Biomarkers are defined as objective indicators of normal biologic processes, pathogenic processes or pharmacologic responses to therapeutic interventions or to an exposure, and have the potential to decrease the length and cost of trials and enrich our understanding of the pathogenesis of a disease [9]. An appropriate biomarker should, therefore, document disease progression or disease activity and should assess any effects of therapeutic intervention [10]. Biomarkers are useful to assess joint tissue turnover, they can reflect the pathological processes due to a joint bleed. Ideally, these biochemical markers should monitor joint status more closely and more accurately than radiography, US and MRI, especially in early phases. In osteoarthritis and rheumatoid arthritis, biomarkers related to the degradation of cartilage, bone and synovial tissue have successfully been studied to assess the severity and progression of joint damage, and to control the effects of treatments [11,12]. Although HA resembles characteristics of both OA and RA, few data on biomarkers in this clinical setting are available.

The aim of this review is to summarize and categorize publications about biomarkers in HA focusing on biochemical markers.

The goal of this review is also to clarify if the biomarkers can be used in the clinical practice to monitor bleeding, to assess the progression of the HA, and to evaluate the effectiveness of the treatments.

## 2. Results

### 2.1. Early Joint Disease

#### 2.1.1. Motion Analysis

Gait analysis is a well-established tool for the quantitative assessment of gait disturbances providing functional diagnosis, assessment for treatment planning and monitoring of disease progress [13]. As highlighted in a recent review [14], gait analysis is a promising tool to detect early walking changes with a non-invasive and well-tolerated examination especially in the pediatric population [15,16,17,18,19,20]. In adulthood, this technique may be also useful to help detect walking worsening in patients with a known diagnosis of arthropathy [21,22,23].

Early alterations in muscle function remain often undetected because the clinical scores usually adopted to examine joint status in patients with hemophilia are able to detect late function impairment when flexion contracture, shortening, weakening or atrophy become evident and rehabilitation is more difficult [14,15,24,25,26,27,28,29].

#### 2.1.2. US and MRI

Joint health is usually evaluated by clinical examinations, clinical scores and imaging. X-rays allow only for the detection of more advanced signs of joint deterioration and are unable to provide information about early changes.

Both MRI and US, with the respective advantages, make it possible to assess early signs of joint disease [2,30].

The results reported above are summarized in Table 1.

### 2.2. Hemarthrosis

#### 2.2.1. Biochemical Markers

Xu el al. in 2020 conducted a study to assess the levels of inflammatory factors and angiogenic factors in patients with severe hemophilia A and to evaluate their diagnostic values for acute joint bleeding. The study included a total of 144 patient with severe hemophilia A and 90 healthy volunteers as a control group. Compared with healthy volunteers, the levels of leukocytes, C-reactive protein (CRP), macrophage migration inhibitory factor (MIF) and vascular endothelial growth factor (VEGF) were significantly (*p* < 0.05) elevated in the patients with severe hemophilia A and were significantly higher in patients with joint bleeding than in nonbleeding patients (*p* < 0.05). They have concluded that CRP and VEGF were independent risk factors for acute joint bleeding (*p* < 0.05), and that levels of inflammatory factors and angiogenesis factors are elevated in patients with severe hemophilia A at baseline and, to a greater degree, after acute joint bleeding [31].

Different results were obtained by Zetterberg and colleagues, they found that microvascular density and VEGF expression were significantly increased in synovial tissue from hemophilic patients compared with controls, they also found that here was no difference in pericyte coverage of synovial vessels or levels of VEGF in plasma, platelet lysates or synovial fluid [32].

Two studies have evaluated the CRP in plasma and in serum providing different results. Karapnar et al. have investigated the relationship between joint findings and levels of serum angiogenic and inflammatory factors in severe hemophilia A patients; the patient groups consisted of 10 severe hemophilia A patients with acute joint bleeding and 25 severe hemophilia A patients without acute joint bleeding, the control group consisted of 22 healthy male children. CRP and MIF levels were detected significantly higher in hemophilia patients with acute joint bleeding than patients without acute joint bleeding. There was a positive correlation between serum TM, VEGF and MIF levels. This study has demonstrated that serum CRP and MIF levels increases in acute bleeding period regardless of the presence of previous joint damage in children with severe hemophilia and that CRP elevation may be a useful and rapid marker for acute bleeding in severe hemophilia A patients [33]. A different work found no strong correlation between CRP level and radiological and physical examination of the joint [34].

Some other studies have investigated the ability of biochemical markers to differentiate a simple bleed from a flare-up of HA. The levels of plasma epidermal growth factor (EGF), colony stimulating factor 2 (CSF2), interleukin 4/13 (IL4/13), fibroblast growth factor 2 (FGF2) and MIP-1 α were significantly lower in patients with a joint bleeding compared to patients without a bleeding or synovitis [35]. The levels of serum D-dimer, ferritin, FDP, leukocyte, plasminogen and VEGF were statistically significantly increased in patients with acute joint bleeding. No clear differences were found in patients with severe hemophilia with and without acute joint bleeding for the serum markers endostatin, ferritin, ICAM-1, lactic acid, thrombomodulin (TM), VEGF and hemoglobin [31,33].

In 2016 Sen et al. have analyzed the role of a microRNA biomarkers (miR-15b) in the development of articular disease in an acute and chronic hemarthrosis model of hemophilia A mice, they found that miR-15b was consistently repressed from the onset of joint bleeding until six bleeding episodes had occurred (so up to 90 days). To test whether reconstitution of miR-15b modulates biomarkers of joint damage in a chronic hemarthrosis model, they administered an adeno-associated virus (AAV) 5-miR-15b vector intra-articularly alone or in combination with systemic administration of AAV2-FVIII. miRNA-15b overexpression has demonstrated to downregulated markers of angiogenesis and hypoxia (VEGF-α and hypoxia inducing factor 2α) in the affected articulations. Moreover, the coadministration of miRNA15b and FVIII vectors diminished the levels of the chondro-degenerative matrix metalloproteinases 1, 3, 9 and 14 in the affected joints [8,36].

The work of Sen et al. provides a basis for the first time to: comprehensively screen miRNAs from the onset of bleeding to the development of arthropathy so as to define miRNA based biomarkers; understand mechanisms behind dysregulation of crucial molecular mediators and their pathogenic contribution to arthritis in a stage-specific manner; and to design miRNAs based therapeutics for minimizing joint disease in hemophilia.

#### 2.2.2. Annual Bleeding Rate (ABR)

To establish the degree of HA is also possible to assess the annual bleeding rate (ABR), which was demonstrated to be directly associated with the degree of arthropathy on X-rays assessed by the Pettersson score (PS) [37], the serum level of the endothelial specific isoform of type XVIII collagen (COL-18N) was significantly associated with the ABR and might be a promising marker [38].

#### 2.2.3. US and MRI

US has proven to be extremely sensitive in detecting low concentrations of intra-articular blood (as low as 5%) and have shown to be helpful either in distinguishing between inflammatory (serous) effusion from hemarthrosis [39] then in defining whether acute pain episodes in hemophilia patients are related to a bleed or to arthritis-mediated conditions [40]. Although joint effusion can be considered an indicator of acute hemarthrosis, this sign is reported as a transitory fluctuating parameter and cannot express the status of a joint [41].

MRI can enable the differentiation of a simple effusion from a hemorrhagic one; unenhanced studies using gradient echo (GRE) sequences can improve the visualization of synovium, cartilage and hemosiderin [42,43].

The results reported above are summarized in Table 2.

### 2.3. Synovitis

#### 2.3.1. Inflammation and Vascular Biomarkers

The inflammation and angiogenesis process in HA were investigated in several studies and can be classified as diagnostic/prognostic biomarkers. VEGF is the principal signaling molecule in angiogenesis. Patients with early joint disease showed a 10-fold increase in plasma VEGF-A compared to patients with advanced joint disease in one study [44]. In contrast, two other studies reported no significant differences for serum or plasma VEGF and different stages of HA [33,45]. Acharya et al. investigated levels of plasma VEGF-A, stromal cell-derived factor 1 alfa (SDF-1α) and matrix metallopeptidase 9 (MMP-9) in hemophilia patients with joint disease versus patients with a bleeding disorder without joint disease and found a significant 4-fold elevation in hemophilia patients [44].

A biomarker to evaluate the severity of hemophilia is plasma soluble vascular cell adhesion protein 1 (VCAM-1), which was significantly higher in patients with more severe arthropathy on X-ray [46].

Among the synovitis biomarkers were also studied: plasma MMP-9, SDF-1a, soluble VCAM-1, serum calprotectin, (high sensitive) CRP, plasminogen, FDP, D-dimer, ferritin and MIF that were significantly increased in hemophilia patients compared to controls, whereas results for plasma leukocyte and serum lactic acid were unclear or contradictory. Plasma hemoglobin (Hb), monocyte, platelet, soluble E-selectin and P-selectin and serum α2-antiplasmin (α2-AP), endostatin, TM and intercellular adhesion molecule-1 (ICAM-1) did not differ between subjects [33,46,47].

The inflammatory markers were investigated in hemophilic mice with and without an induced joint bleeding by Haxaire et al. in 2017, their work was based on the assumption that joint bleedings can promote a systemic proinflammatory condition and that, consequently, inflammatory markers could be a suitable biomarker for the detection of hemarthrosis. The results have demonstrated that serum calprotectin, a marker for residual inflammation, was higher in the hemarthrosis induced mice at two and twelve weeks compared with control hemophilic mice, where calprotectin was not detectable [47].

#### 2.3.2. US and MRI

Several studies consistently showed comparable sensitivity between US and MRI imaging for the detection of synovial hypertrophy [30,48,49,50].

With Doppler imaging, US has proved able to detect synovial hyperemia [51,52,53].

Contradictory results are reported in the literature on the ability of US to detect intra-articular deposition of hemosiderin. Some authors described some distinctive echo textural features between hemosiderin and synovium [41,49,53,54] but other authors did not find any difference between the US appearance of hemosiderin-laden and hemosiderin-free synovium [55,56,57].

MRI imaging is a sensitive technique to visualize hemosiderin deposition in a joint, especially using T2* GRE sequences [58,59].

The results reported above are summarized in Table 3.

### 2.4. Cartilage Degeneration

#### 2.4.1. Cartilage Turnover Markers

The cartilage turnover markers can be classified as diagnostic biomarkers markers. Urinary C-telopeptide fragments of type II collagen (CTX-II) was studied once and showed a significant correlation with joint space narrowing (JSN) on the X-ray and the total radiographic Pettersson score (PS). [60] One study has found a significant correlation for serum chondroitin sulfate epitope 846 (CS846) and serum C1, 2C with JSN; in this study was used combined index to increase correlations of single biomarkers indeed the combined index of uCTX-II and serum C1, 2C and CS846 increased the correlation with JSN and PS [60]. On the contrary, a different study found no strong correlation between the same biomarkers and radiological and physical examination of the joint [34]. Oldenburg et al. did not confirm the correlations for serum CS846 and X-ray parameters, on the contrary they found a significant correlation of serum CS846 with MRI score in a small subpopulation [45]. The relationship of serum cartilage oligomeric matrix protein (COMP) with arthropathy was evaluated in five studies with different results. Two studies reported a significant positive association of COMP with JSN on the X-ray while three other studies reported weak negative or no correlations with the severity of HA on the imaging [34,45,60,61,62]. The serum level of COMP was studied in three different studies with contradictory results. Two independent studies conducted by Hassab et al. in 2016 and Hua et al. in 2017 [34,61] reported a significantly higher level of COMP in patients with HA compared to healthy controls; a conference abstract by Sun et al. reported that levels of serum COMP was both significantly lower in adult patients with severe hemophilia A than in controls [62].

Pulles et al. have evaluated eight biomarkers including urinary CTX- II, serum C1, 2C, CS-846 and COMP in 36 hemophilia patients, with various degrees of HA, that were followed-up for a mean of 6.5 years. Neither of the individual biomarkers measured at baseline predicted the progression of joint damage. However, the combined index of urinary CTX-II and serum CS846 was significantly associated with radiographic joint damage progression (odds ratio 8.8, 95% confidence interval: 1.1-70.6 *p* = 0.04). The discriminative ability of the prediction model of the combined index was considered “acceptable” with an area under the curve of 0.77 (95% CI: 0.60-0.95). This explorative study demonstrated that the combination of urinary CTX-II (a biochemical marker representing degradation) and serum CS846 (a biochemical marker representing synthesis) has the potential to predict radiographic progression of joint damage in HA, on the other side neither of the individual biomarkers by itself showed a prognostic value, this could be a reflection of the complexity of the different process contributing to joint damage after blood exposure [63].

A conference abstract has evaluated—in 30 patients with hemophilia and HA—if the level of cartilage turnover biomarkers increased and if the level could be compared with the severity of the HA. They collected serum from 30 severe hemophilia A adult patients with severe arthropathy and 19 normal control subjects with age- and gender-matched. The results showed that the levels of serum type II collagen degradation (sColl2-1) and COMP were both significantly lower in adult patients with severe hemophilia A than in controls, the concentrations of cartilage turnover biomarkers in patients with on-demand treatment were lower than prophylaxis, despite being without significant differences. They have also observed a weak correlation between the cartilage turnover biomarkers and ultrasound score and relatively strong correlations between the change of cartilage turnover biomarkers and ultrasound score change. These results indicate that levels of cartilage turnover biomarkers might change in hemophilia patients with progression of joint structure damage [62].

In 2016, Manon-Jensen et al. used serological biomarkers of collagen turnover to evaluate remodeling of the extracellular matrix in a model of hemophilic arthropathy of rats with factor VIII deficiency. Rats with factor VIII deficiency and wild-type littermate controls were subjected to repeated knee bleedings induced by needle puncture on days 0 and 14. Collagen turnover biomarkers were studied, including collagens of the basement membrane (type IV collagen), interstitial matrix (collagen types III, V and VI) and cartilage (type II collagen). In rats with factor VIII deficiency the serum level of MMP-degraded collagen type 2 marker (C2M), that is a collagen type II degradation marker, did not change after the first bleeding but they significantly increased after the second joint bleeding and the serum levels of C2M after the second joint bleed significantly correlated with the degree of arthropathy on histology. Additionally, other collagen markers like serum MMP-degraded collagen type 4 marker (C4M) and 7 S domain of type 4 collagen (P4NP7S) increased significantly one day after the second joint bleeding, whereas serum MMP-degraded collagen type 3 marker (C3M) significantly decreased directly after the joint bleeding, interestingly serum pro-peptide of type 5 collagen (PRO-C5) and procollagen type 3 N-terminal peptide (P3NP) increased significantly one week after the first and second joint bleeding. The main conclusion was that in hemophilic rats, joint hemorrhage and hemophilic arthropathy cause changes in the turnover of extracellular matrix collagens [8,64].

A different work has demonstrated that, in hemophilic mice, plasma levels of C4M and pro-peptide of type 4 collagen (PRO-C4) were significantly increased two weeks after an induced hemarthrosis [65].

In 2015 van Vulpen et al. have experimentally induced hemarthrosis in dogs to evaluate the change of urinary CTX-II and serum COMP, CS846 and C1, 2C in response to a joint bleed, the study has demonstrated a significant increase in urinary CTX-II from day two to seven (from 75% to 155%) and serum COMP from baseline to day two (+46%). In the same study van Vulpen et al. have evaluated whether biomarkers of joint damage were sensitive to change shortly after hemarthrosis also in patients with hemophilia. They collected blood and urine samples from ten patients with hemophilia who had reported experiencing a joint hemorrhage: at 2 days, at 3–5 days and at 12–14 days. To know the value the baseline condition of the biomarker, 90 days after the hemorrhage, a blood and urine sample were collected from the patients. Commercial serum and urine biomarker assays were performed: urinary CTX-II, serum COMP, C1, 2C and serum CS846. In patients with hemophilia, the levels of urinary CTX-II and serum CS846 increased five days after the joint hemorrhage comparing to the initial value. These results have demonstrated that an increase in biochemical markers of joint tissue turnover can be detected directly after a single joint bleed in patients with varying levels of (hemophilic) arthropathy as well as in a canine model of blood-induced joint damage without pre-existing joint damage [8,66].

Some studies focused on the evaluation of the cartilage formation markers, unfortunately the values of the cartilage formation markers were comparable in patients with HA and in healthy controls with the exception for the marker serum PRO-C5, which was significantly downregulated in hemophilia patients; instead regarding the cartilage degradation markers some were significantly increased in hemophilia patients compared to controls (C2M, CTX-II and C4M), while other markers were significantly decreased (A disintegrin and metalloproteinase with thrombospondin motifs 5—ADAMTS5, Coll2-1, ADAMTS-degraded collagen type 3—C3A, C3M and MMP-degraded collagen type 5 marker—C5M) in hemophilia patients [34,62,67].

Sun et al. have investigated the efficacy of treatment on biochemical markers. The data reported by Sun et al. suggested that patients treated on-demand had lower cartilage turnover markers compared with patients treated on a prophylactic basis though no statistical significance could be demonstrated in the study due to the small size of the sample [62].

#### 2.4.2. US and MRI

In the US-beam accessible areas, US has proved to be very sensitive to detect abnormalities of the articular cartilage and subchondral bone, even if arthropathy is initial and still localized, with a spatial resolution even higher than surface-coiled MR imaging [2,68].

MRI is still the best imaging modality for the detailed evaluation of osteochondral derangements in HA, particularly those pertaining to the central aspect of the cartilage and subchondral bone evaluation [40,49,54,69,70,71].

The results reported above are summarized in Table 4.

### 2.5. Bone Involvement

#### 2.5.1. Bone Turnover Markers

Considering the bone turnover markers, the most investigated were C-terminal telopeptide of type 1 collagen (CTX-I) and serum osteocalcin. CTX-I was studied in six different studies with heterogeneous results, one study showed a weak but significant association with the degree of arthropathy on X-ray, while the other studies reported non-significant relations [34,45,60,72,73].

Serum osteocalcin was evaluated in four studies with contradictive results, one study reported a significant negative associations of serum osteocalcin with the physical examination score CHPJPES, while three other studies did not report significant associations between serum osteocalcin and HJHS or PS [72,73,74,75].

Serum tartrate-resistant acid phosphatase 5b (TRAP-5b) was investigated in one study, serum sclerostin was investigated in two studies, both these bone turnover markers had a significant correlation with the severity of HA [72,73,76]. Serum Dickkopf-1 (Dkk-1), vitamin D and bone alkaline phosphatase (b-ALP) were significantly correlated with physical examination scores but not with imaging scores/number of affected joints [72,73,75,76]. Correlations of serum osteoprotegerin and receptor activator of nuclear factor kappa-B ligand (RANK-L) were contradictory [73,75].

Hua et al. have investigated whether biomarkers of cartilage and bone degradation, and inflammation were altered in patients with hemophilia and whether these biomarkers could identify hemophilia patients with arthropathy. In this study, using a biomarker panel combining C2M, C-reactive protein metabolite (CRPM) and ADAMTS5 they could distinguish hemophilia patients from control subjects with 85.3% accuracy (*p* < 0.0001), unfortunately they found no strong correlation between biomarkers and radiological and physical examination of the joint [34].

#### 2.5.2. X-ray, US and MRI

Synovial hypertrophy, inflammation, joint effusion, hemosiderin deposits and early cartilage damage are not clearly delineated on plain radiographs and can appear as nonspecific soft tissue swelling on radiography [42].

Compared to radiography, US has demonstrated higher sensitivity to detect early damage signs [53]. Good correlation was observed between US and MRI imaging in the evaluation of bone erosions and cartilage abnormalities in the elbows, knees and ankles [48,50]. Muca-Perja et al. adopted a semiquantitative scoring system to quantify joint changes at ultrasonography [2,5,42,49].

MRI provides detailed information about joint status allowing additional assessment of early bone lesions. In the largest study comparing plain radiographs with MRI findings, chronic synovitis was demonstrated in 50% of patients in whom the plain radiograph was reported as normal. In those that did demonstrate plain film abnormalities, MRI revealed more profound disease in 70% [77]. Subchondral oedema and cysts may be associated with high-intensity signal on fluid-sensitive sequences [2]. Considering the international MRI scale, the Denver scale is based on the single most severe MRI finding and the European score values bone and soft damages [42]. In 2012 the International Prophylactic Study Group (IPSG) merges the two scores into a single scale, which proved useful for the analysis of both early and moderate stages of arthropathy [40].

The results reported above are summarized in Table 5.

## 3. Discussion

Early joint damage in patients with hemophilia often escapes diagnosis because of insufficient investigation of biomechanical changes. Biomechanical reactions of muscles and ligaments around the joint have been less investigated, although similarly important, than the biochemical reactions linked to blood-induced inflammation and damage of joint tissues. Furthermore, the biomechanical reactions are the first to occur immediately after bleeding, as muscles react with changes in contraction pattern and ligaments overloading as demonstrated by the gait analysis [21] and surface electromyography (sEMG) [25].

As function impairment usually precedes structural damage [25], it is of major importance to include more sensitive measurement tools for a more accurate musculoskeletal examination of patients with hemophilia. The early diagnosis may facilitate the initiation of appropriate joint-based therapeutic concepts and should trigger the implementation of training programs in order to preserve hemophilic joints from fast deteriorating. Moreover, the motion analysis by means of sEMG and/or gait analysis can be also useful to evaluate the patient’s response to treatment.

The subtle articular changes of the subclinical disease can be detected by diagnostic imaging techniques. The detection of early signs of osteochondral damage is often difficult. Moreover osteochondral damage can be present in asymptomatic patients in which none or just a few bleeding episodes were previously recognized [30].

Conventional radiography has been successfully used for decades to objectively evaluate HA. Findings of HA on plain radiographs include osteoporosis, osteonecrosis, epiphyseal overgrowth, widening of the intercondylar notch of the distal femur, bone cysts, joint space irregularity and narrowing, angulation of the knee and ankle and bony fusion. However, all these signs represent late arthropathic changes, most notably subchondral and bony abnormalities, and are not useful for an early diagnosis of arthropathy. All these abnormalities are included into the main classification systems, the Arnold–Hilgartner scale and the Pettersson score [49,59].

MRI is the gold standard imaging technique to evaluate the musculoskeletal system because of its excellent spatial and contrast resolution. MRI is the most complete imaging technique and the most sensitive for the diagnosis of musculoskeletal complications of hemophilia; this image technique has shown its interest to detect early signs in joint alteration and it has been shown to be more sensitive in detecting the first signs of HA than both clinical examination and plain radiography [78].

The signal of blood products on MRI varies according to the sequence used. Susceptibility-sensitive T2n GRE techniques result in enhanced visibility of blood products in the acute stage (deoxyhemoglobin) and the chronic stage (hemosiderin).

MRI imaging is a sensitive technique to visualize hemosiderin deposition in a joint, especially using T2* GRE sequences [58,59].

If susceptibility artifact from hemosiderin limits interpretation, GRE sequences replaced with T2-weighted tSE sequences is a suitable alternative [79].

On proton density sequences or 3D spoiled gradient echo (GRE), chronic synovial proliferation is characterized by intermediate intensity signal on T1 and T2-weighted sequences presenting a level of contrast between cartilage and fluid [58]. However, in the active synovitis phase, the MRI signal intensity of the proliferating synovium may increase becoming similar to the fluid one so that the distinction with effusion could be difficult [80].

The use of intravenous contrast media may theoretically help distinguish active synovitis from fibrotic synovium [43]. However, the presence of hemosiderin and synovium within the proliferative synovium in HA limits the degree of visible enhancement; for these reasons intravenous contrast medium is not routinely recommended in the evaluation of HA [42].

In terms of clinical relevance, detection of synovial hypertrophy, regardless of its degree of detectable vasculature, represents a sign of undertreatment, possibly related to an insufficient treatment regimen or a limited patient’s compliance [41].

MRI is also the best imaging modality for the detailed evaluation of osteochondral derangements in HA, particularly those pertaining to the central aspect of the cartilage and subchondral bone evaluation [40,49,54,69,70,71].

Detailed imaging of the articular cartilage obtained with either a proton density fat-suppressed or volumetric GRE sequences may demonstrate focal and diffuse cartilage losses, whereas subchondral edema and cysts may be associated with high-intensity signal on fluid-sensitive sequences [2].

MRI remains difficult to use in routine clinical practice because of its cost and limited accessibility [42].

US has a low cost, short examination time and it is widely accessible. With the advent of the last generation equipment, by US it is now possible to depict small, superficial structures of the musculoskeletal system as present in the early stages of hemophilic arthropathy [30].

US has proved able to detect synovial hyperemia, defined as intrasynovial detection of blood flow signals [51,52,53]. However, intrasynovial hyperemia at Doppler imaging is uncommonly observed in hemophilic patients and, in the rare positive cases, only a few blood flow signals are visualized, suggesting mild hypervascularity that cannot be considered relevant enough to redirect treatment and patient management [69]. In addition, high variability in the interpretation of Doppler images, the need for high-end machines to get better performance and high interequipment variability is expected [41].

Regarding osteochondral surfaces, US cannot provide a comprehensive evaluation of the cartilage and subchondral bone like MRI can do.

Indeed, the weight-bearing areas, cannot be clearly assessed due to the problem of the access of the US beam [48,49,54].

In regard to the articular cartilage, US is able to detect the full spectrum of abnormalities, from subtle echo textural changes or partial thickness losses through extensive cartilage disappearance. In children, coexisting damage of the epiphyseal cartilage can be recognized [2].

Although clinical examination and radiological exams (radiographs, US and MRI) remain the gold standard for the diagnosis of hemophilic arthropathy and hemarthrosis in patients with hemophilia, serological biomarkers of bleeding and joint degeneration can be an attractive complement to the diagnostic imaging techniques.

In our review we pointed out that, at the present time, biomarkers are interesting scientifically intriguing, but they are not standardized for the use in clinical practice. Attempts to investigate the role of the biomarkers in the diagnosis of hemophilic arthropathy has yielded conflicting results, because of different inclusion or exclusion criteria adopted by the different studies and/or classification of HA.

Biomarkers for monitoring articular bleeding and consecutive progressive cartilage destruction in hemophilic patients would be a useful tool for the clinician, but further studies are needed to confirm and develop the present knowledge on existing biomarkers and hopefully identify new, more specific ones.

Even though the use of biomarkers such as miRNA in the screening or follow up of this condition is still limited to the animal model studies, they seem to lay a promising basis for future use in the clinic.

The inflammation and angiogenesis process in HA were investigated in several studies and can be classified as diagnostic/prognostic biomarkers. VEGF is the principal signaling molecule in angiogenesis and can be induced by hypoxia and by certain cytokines through interaction with its receptors. Previously research has demonstrated that the synovial reaction in other joint diseases—that share histologic similarities with HA—enhances oxygen demand and shows evidence of de novo blood vessel formation, including endothelialization of the synovium. Importantly, proliferating synovium can secrete chemocytokines, such as VEGF, that might promote recruitment of endothelial cells to sites of active angiogenesis [81]. Building on this knowledge the changes in VEGF values have been analyzed in several studies with contradictive results. VEGF was found to be increased in HA patients with early joint disease, but other studies obtained contrasting results; SDF-1a and MMP-9 were also investigated and found to be increased in HA patient with joint disease; a correlation between VCAM-1 and severe arthropathy at X-ray was identified in a study and other ones found an association of CRP and MIF with acute joint bleeding in HA patients. Considering the contrasting evidence regarding the sensitivity and specificity of the biomarkers, the detection of arthropathy in HA patients still relies on imaging techniques: for instance, MRI remains the gold standard for the detection of synovitis in HA patients.

The cartilage turnover markers can be classified as diagnostic biomarkers markers. Type II collagen is the major component of the articular cartilage matrix and is degraded by proteolytic enzymes secreted by chondrocytes and synoviocytes [81]. Several markers of joint tissue turnover and formation were investigated with contradictive results. Some studies have found a correlation between the increase of collagen turnover markers and the increase of JSN and PS, unfortunately another study did not confirm this correlation [34,45,60,61,62].

The different results may be explained by heterogeneous assessment for HA or by different populations; for all these reasons comparison between studies is often difficult.

When it comes to bone turnover, results on the use of CTX-I, serum osteocalcin, serum osteoprotegerin and RANK-L are quite heterogeneous, while there seem to be a correlation between the level of serum TRAP-5 and serum sclerostin with the severity of the HA. On the other hand, serum DKK-1, vitamin D and b-ALP were significantly correlated with physical exam findings, but not radiological ones. However, further studies are needed to support their use in the clinical practice.

## 4. Materials and Methods

A review of the literature was performed on two medical electronic databases, PubMed/MEDLINE and Embase, from 3 to 7 August 2020. The study selection and the data extraction were achieved independently by two authors, meanwhile the senior investigators revise the work. Inclusion criteria were determined a priori:

- all studies were written in English;
- all studies have available full text;
- all studies were published in peer-reviewed journals.

We excluded studies not reporting data concerning hemophilic disease.

Twenty (20) articles not meeting the inclusion criteria were identified by checking the bibliography of relevant articles and searching for the studies cited in all the articles examined.

Eligible studies obtained at the end of the search and screen process were seventy-three (73).

The search string in PubMed/MEDLINE for biochemical biomarkers was:

- Hemophilia biomarkers AND arthropathy.

The search produced forty-six (46) articles.

Two different Embase searches for biochemical biomarkers were performed using two different strings as follows:

- (‘hemophilia’/exp OR ‘hemophilia’) AND (‘biomarkers’/exp OR biomarkers) AND (‘arthropathy’/exp OR ‘arthropathy’).

The search produced eighty-three (83) articles.

- (‘hemophilia’/exp OR ‘hemophilia’) AND (‘biological markers’/exp OR ‘biological markers’)

Meanwhile the second search produced two hundred eleven (211) articles.

The two different searches in Embase had in common fifty-eight (58) articles and all three different searches (PubMed/MEDLINE and Embase) had in common thirteen (13) articles.

Finally, fifteen (15) articles were selected.

Regarding the evaluation of imaging techniques, such as radiographs, ultrasound and MRI, a full text articles research on PubMed/MEDLINE database, considering the last 5 years, was performed using the string as follow:

- Hemophilia arthropathy imaging.

The research produced one hundred and twenty-four (124) articles.

In Embase a search was performed using the follow string, limiting the research to the studies published in the last 5 years:

- ‘hemophilia ultrasound mri’ OR ((‘hemophilia’/exp OR hemophilia) AND (‘ultrasound’/exp OR ultrasound) AND (‘mri’/exp OR mri)).

The research produced seventy-two (72) articles.

Seventeen (17) articles were shared between Embase and PubMed research.

The articles selected were twenty-two (22).

The research for the motion analysis was performed in PubMed/MEDLINE and in Embase selecting full text articles in the last 5 years. The PubMed string was:

- Hemophilic arthropathy gait analysis;

and the Embase string was:

- ‘hemophilia arthropathy gait analysis’ OR ((‘hemophilia’/exp OR hemophilia) AND (‘arthropathy’/exp OR arthropathy) AND (‘gait’/exp OR gait) AND (‘analysis’/exp OR analysis)).

The research in PubMed/MEDLINE produced nine (9) articles and the research in Embase thirty-eight (38) articles. The two databases had in common seven (7) articles and for this review after reading the texts were selected sixteen (17) articles.

Unfortunately, a meta-analysis was not performed because the studies showed variability in test measures.

## 5. Conclusions

Recurrent hemarthroses lead to specific and reversible changes in the synovium and cartilage, which finally result in the destruction of the joint. A problem continues to exist in our ability to detect hemarthrosis sequela at the preclinical or asymptomatic phase, when the disease process is early and potentially reversible. The clinical use of biomarkers could be extremely helpful in early diagnosis and scientific research underlined how interest is very much on the rise. Nevertheless, the clinical use of biomarker still has little bearing on clinical practice because of the lack of standardization. In this sense, in our opinion, the standardization of biomarkers through their association with clinical and radiological parameters will be promising.

## Figures and Tables

**Table 1:** Markers related to the early joint disease stage.

Early Joint Disease	
Motion Analysis	
**Gait Analysis**	
**sEMG**	surface electromyography
**Imaging**	
**US**	ultrasound
**MRI**	magnetic resonance imaging

**Table 2:** Markers related to hemarthrosis stage and their modifications.

Hemarthrosis			
Biochemical Markers			
**VEGF**	**vascular endothelial growth factor**	blood protein	↑
**CRP**	C-reactive protein	blood protein	↑
**MIF**	phagocyte migration inhibitory factor	blood protein	↑
**D-dimer**		blood protein	↑
**ferritin**		blood protein	↑
**FDP**	fibrin/fibrinogen degradation products	blood protein	↑
**leukocyte**		blood cells	↑
**plasminogen**		blood protein	↑
**EGF**	epidermal growth factor	blood protein	↓
**CSF2**	colony stimulating factor 2	blood protein	↓
**IL4/13**	interleukin 4/13	blood protein	↓
**FGF2**	fibroblast growth factor 2	blood protein	↓
**MIP-1 α**	macrophage inflammatory protein 1-alpha	blood protein	↓
**miR-15b**	microRNA 15b	tissue microRNA	↓
**TM**	thrombomodulin	blood protein	↑
**ABR**	Annual bleeding rate		↑
**COL-18N**	endothelial specific isoform of type XVIII collagen	blood protein	↑
**Imaging**			
**US**	ultrasound		
**MRI**	magnetic resonance imaging		

**Table 3:** Markers related to synovitis stage and their modifications.

Synovitis and Synovial Hypertrophy			
Biochemical Markers			
**VEGF**	**vascular endothelial growth factor**	blood protein	↑
**SDF-1α**	stromal cell-derived factor 1 alfa	blood protein	↑
**MMP-9**	matrix metallopeptidase 9	blood protein	↑
**VICAM-1**	vascular cell adhesion protein 1	blood protein	↑
**Calprotectin**		blood protein	↑
**CRP**	C-reactive protein	blood protein	↑
**plasminogen**		blood protein	↑
**FDP**	fibrin/fibrinogen degradation products	blood protein	↑
**D-dimer**		blood protein	↑
**ferritin**		blood protein	↑
**MIF**	phagocyte migration inhibitory factor	blood protein	↑
**Hb**	hemoglobin	blood protein	=
**monocyte**		blood cells	=
**platelet**		blood component	=
**E-selectin**		blood protein	=
**P-selectin**		blood protein	=
**α2-AP**	α2-antiplasmin	blood protein	=
**endostatin**		blood protein	=
**TM**	thrombomodulin	blood protein	=
**ICAM-1**	intercellular adhesion molecule - 1	blood protein	=
**Imaging**			
**US**	ultrasound—Doppler imaging		
**MRI**	magnetic resonance imaging		

**Table 4:** Markers related to cartilage degeneration stage and their modifications.

Cartilage Degeneration			
Biochemical Markers			
**U-CTX-II**	**urinary C-telopeptide fragments of type II collagen**	urinary protein	↑
**CS846**	chondroitin sulfate epitope 846	blood protein	↑
**C1**	serum cartilage cleavage products	blood protein	↑
**2C**	serum cartilage cleavage products	blood protein	↑
**COMP**	serum cartilage oligomeric matrix protein	blood protein	↑/↓
**Coll2-1**	type II collagen degradation	blood protein	↓
**C2M**	MMP-degraded collagen type 2 marker	blood protein	↑
**C3M**	MMP-degraded collagen type 3 marker	blood protein	↓
**C4M**	MMP-degraded collagen type 4 marker	blood protein	↑
**C5M**	MMP-degraded collagen type 5 marker	blood protein	↓
**P4NP7S**	7 S domain of type 4 collagen	blood protein	↑
**PRO-C4**	pro-peptide of type 4 collagen	blood protein	↑
**PRO-C5**	pro-peptide of type 5 collagen	blood protein	↑
**P3NP**	procollagen type 3 N-terminal peptide	blood protein	↑
**C3A**	ADAMTS-degraded collagen type 3	blood protein	↓
**ADAMTS5**	A disintegrin and metalloproteinase with thrombospondin motifs 5	blood protein	↓
**Imaging**			
**X-ray**	joint space narrowing (JSN), Pettersson Score (PS)		
**US**	ultrasound		
**MRI**	magnetic resonance imaging		

**Table 5:** Markers related to the bone involvement stage and their modifications in hemophilic arthropathy (HA).

Bone Involvement			
Biochemical Markers			
**CTX-1**	**C-terminal telopeptide of type 1 collagen**	blood protein	↑/=
**osteocalcin**		blood protein	↑/=
**TRAP-5b**	tartrate-resistant acid phosphatase 5b	blood protein	↑
**sclerosant**		blood protein	↑
**Dkk-1**	dickkopf-1	blood protein	↑
**Vitamin D**		blood protein	↑
**b-ALP**	bone alkaline phosphatase	blood protein	↑
**osteoprotegerin**			
**RANK-L**	receptor activator of nuclear factor kappa-B ligand		
**C2M**	MMP-degraded collagen type 2 marker	blood protein	↑
**CRPM**	C-reactive protein metabolite	blood protein	↑
**ADAMTS5**	A disintegrin and metalloproteinase with thrombospondin motifs 5	blood protein	↓
**Imaging**			
**X-ray**	joint space narrowing (JSN), Pettersson Score (PS), Arnold-Hilgartner scale		
**US**	Muca-Perja et al. scoring system		
**MRI**	Denver scale, European score, International Prophylaxis Study Group (IPSG) scale		

## Abbreviations

2C	serum cartilage cleavage products
ABR	annual bleeding rate
ADAMTS5	a disintegrin and metalloproteinase with thrombospondin motifs 5
b-ALP	bone alkaline phosphatase
C1	serum cartilage cleavage products
C2M	MMP-degraded collagen type 2 marker
C3A	ADAMTS-degraded collagen type 3
C3M	MMP-degraded collagen type 3 marker
C4M	MMP-degraded collagen type 4 marker
C5M	MMP-degraded collagen type 5 marker
COL-18N	endothelial specific isoform of type XVIII collagen
Coll2-1	type II collagen degradation
COMP	serum cartilage oligomeric matrix protein
CRP	C-reactive protein
CRPM	C-reactive protein metabolite
CS846	chondroitin sulfate epitope 846
CSF2	colony stimulating factor 2
CTX-1	C-terminal telopeptide of type 1 collagen
Dkk-1	dickkopf-1
EGF	epidermal growth factor
FDP	fibrin/fibrinogen degradation products
FGF2	fibroblast growth factor 2
FIX	factor IX
FVIII	clotting factor VIII
HA	hemophilic arthropathy
Hb	hemoglobin
ICAM-1	intercellular adhesion molecule - 1
IL4/13,	interleukin 4/13
MIF	phagocyte migration inhibitory factor
MIP-1 α	macrophage inflammatory protein 1-alpha
miR-15b	microRNA 15b
MMP-9	matrix metallopeptidase 9
MRI	magnetic resonance imaging
OA	osteoarthritis
P3NP	procollagen type 3 N-terminal peptide
P4NP7S	7 S domain of type 4 collagen
PRO-C4	pro-peptide of type 4 collagen
PRO-C5	pro-peptide of type 5 collagen
RA	rheumatoid arthritis
RANK-L	receptor activator of nuclear factor kappa-B ligand
SDF-1α	stromal cell-derived factor 1 alfa
sEMG	surface electromyography
TM	thrombomodulin
TRAP-5b	tartrate-resistant acid phosphatase 5b
U-CTX-II	urinary C-telopeptide fragments of type II collagen
US	ultrasound
VEGF	vascular endothelial growth factor
VICAM-1	vascular cell adhesion protein 1
α2-AP	α2-antiplasmin
